# Impact of Genetic Variants on Vitamin E Levels in an Italian Cohort of Bariatric Surgery Patients: A Focus on SNPs Involved with Transport and Bioavailability

**DOI:** 10.3390/ijms26020651

**Published:** 2025-01-14

**Authors:** Claudia Ricci, Annalisa Bufano, Gabriele Iraci Sareri, Maria Simon Batzibal, Carlotta Marzocchi, Giorgia Simoncelli, Delia Righi, Antonia Salvemini, Cristina Ciuoli, Leonardo Di Stefano, Nicoletta Benenati, Tommaso Regoli, Kateryna Miedviedieva, Andrea Tirone, Costantino Voglino, Selenia Pirisinu, Silvia Cantara

**Affiliations:** 1 Department of Medical, Surgical and Neurological Sciences, University of Siena, 53100 Siena, Italy; claudia.ricci@unisi.it (C.R.); annalisa.bufano2@unisi.it (A.B.); g.iracisareri@student.unisi.it (G.I.S.); simoncelligiorgia1@gmail.com (G.S.); d.righi4@student.unisi.it (D.R.); antonia.salvemini@student.unisi.it (A.S.); c.ciuoli@ao-siena.toscana.it (C.C.); l.distefano@student.unisi.it (L.D.S.); nicoletta.benenati@gmail.com (N.B.); tommaso.regoli@gmail.com (T.R.); kate018535@gmail.com (K.M.); 2 Laboratory of Clinical and Translational Research, University Hospital of Siena, 53100 Siena, Italy; carlotta.marzocchi@ao-siena.toscana.it; 3 Unit of Bariatric Surgery, Department of General and Specialized Surgery, University of Siena, 53100 Siena, Italy; andrea.tirone@ao-siena.toscana.it (A.T.); costantino.voglino@unisi.it (C.V.); selenia.pirisinu@ao-siena.toscana.it (S.P.)

**Keywords:** bariatric surgery, vitamin E, polymorphisms

## Abstract

Obesity is a global epidemic associated with chronic inflammation, oxidative stress, and metabolic disorders. Bariatric surgery is a highly effective intervention for sustained weight loss and the improvement of obesity-related comorbidities. However, post-surgery nutritional deficiencies, including vitamin E, remain a concern. This study investigates the role of single-nucleotide polymorphisms (SNPs) in genes related to vitamin E transport and bioavailability in determining vitamin E levels post bariatric surgery. A cohort of 140 patients with obesity undergoing bariatric surgery was analyzed. Serum vitamin E levels were measured before and one year after surgery, and SNPs in genes associated with vitamin E transport and metabolism were genotyped using PCR, DHPLC, and sequencing methods. Associations between SNPs, haplotypes, and vitamin E levels were statistically evaluated. Significant associations were observed between the APOE rs7412 SNP and serum vitamin E levels. The rare T allele was linked to lower vitamin E levels post surgery, with an increased frequency in patients with severe deficiency (<11.6 μmol/L). Haplotype analysis of APOE revealed that the ε2 haplotype (T-T) was strongly associated with vitamin E deficiency. Other SNPs, including CD36 rs1761667, SCARB1 rs4238001, and ABCA1 rs4149314, were also linked to changes in vitamin E levels, suggesting that an impaired bioavailability and transport can be the reason for low vitamin E levels post surgery. Genetic polymorphisms in APOE, CD36, SCARB1, and ABCA1 significantly influence vitamin E status after bariatric surgery. These findings highlight the importance of personalized supplementation strategies considering patients’ genetic profiles to mitigate the risk of vitamin E deficiency and related complications.

## 1. Introduction

Obesity, a global epidemic with rising prevalence, is characterized by an excessive accumulation of adipose tissue, leading to a chronic state of low-grade inflammation and oxidative stress [1]. This proinflammatory background is implicated in the development of insulin resistance, dyslipidemia, metabolic abnormalities (type 2 diabetes), and cardiovascular diseases [1]. Despite intensive efforts to address lifestyle factors contributing to obesity, a substantial proportion of individuals face significant challenges in achieving and maintaining significant weight loss through traditional interventions alone. In this context, bariatric surgery has emerged as a therapeutic approach, offering a robust and sustained means of weight reduction with significant improvements observed in insulin sensitivity, glycemic control, and cardiovascular risk factors. Bariatric surgery encompasses a range of procedures designed to induce weight loss by altering the anatomy of the gastrointestinal tract [2]. Common techniques include gastric bypass, sleeve gastrectomy, and adjustable gastric banding. Bypass surgery involves creating a smaller stomach pouch and rerouting the digestive tract to bypass a portion of the small intestine [2]. While this procedure can lead to significant weight loss, patients undergoing gastric bypass surgery require careful follow-up to monitor their nutritional status and overall well-being. Vitamin and mineral deficiencies are common concerns following gastric bypass surgery, and vitamin E is among the nutrients that require special attention in the postoperative period [3]. Vitamin E is a fat-soluble antioxidant that plays a crucial role in protecting cells from oxidative damage [4]. After surgery, patients are at an increased risk of vitamin E deficiency and may be advised to take supplements to meet their daily requirements. However, the response to vitamin E supplementation differs among individuals, some reaching target levels rapidly, others remaining at critical low levels. The reasons for this variability can be linked to polymorphisms in genes [5] involved with vitamin E bioavailability like Niemann–Pick Disease, Type C1 (NPC1), Cluster determinant 36 (CD36), Scavenger Receptor Class B Member 1 (SCARB1), and ATP Binding Cassette Subfamily A Member 1 (ABCA1), or vitamin E transport like Apolipoprotein B (APOB), Apolipoprotein A5 (APOA5), and Apolipoprotein E (APOE). In this paper, we evaluated the genetic signature of these genes in patients with obesity and correlated vitamin E levels after bariatric surgery with the presence/absence of specific SNPs.

## 2. Results

All SNPs were within the Hardy–Weinberg equilibrium in all patient groups (*p* > 0.05), except for APOB rs1713222 and APOE rs7412 (*p* = 0.040 and *p* = 0.015, respectively) in the group of patients with serum vitamin E levels < 30 μmol/L.

The analysis was first performed by stratifying patients into two groups based on serum vitamin E levels (<30 μmol/L, >30 μmol/L), as 30 μmol/L is considered the threshold required to have beneficial effects on human health [6]. Statistically significant differences in genotype and allele distributions between the two groups of patients were observed for the APOE rs7412 SNP (Table 1). The differences were statistically significant at the genotype (*p* = 0.012) and allele levels, assuming additive, dominant, and recessive models (*p* = 0.002, odds ratio = 5.67; *p* < 0.0001, odds ratio = 5.06; and *p* = 0.018, odds ratio = 1.04, respectively).

Furthermore, statistical analysis showed significant differences only at the allele level for SCARB1 rs11057830 (*p* = 0.039, OR = 1.69), assuming a dominant model, and for CD36 rs1761667 and rs1527479 (*p* = 0.003, OR = 2.33 and *p* = 0.008, OR = 2.09, respectively), assuming a recessive model of inheritance.

Considering that the vitamin E levels in the group of patients with serum concentrations <30 μmol/L were quite heterogeneous, we further divided these patients into two subgroups with vitamin E levels between 11.6 and 30 μmol/L or <11.6 μmol/L, as 11.6 μmol/L is the functional deficiency threshold [6]. Again, statistically significant differences among the three groups of patients were observed for the APOE rs7412 SNP (Table 2) at the genotype (*p* = 0.008) and allele levels, assuming additive, dominant, and recessive models (*p* = 0.002, *p* < 0.0001, and *p* = 0.002, respectively). In all cases, the percentage of the T allele increased progressively as serum vitamin E levels decreased. For example, considering the additive model, the frequency of the T allele was 2.3% in patients with vitamin E levels > 30 μmol/L, 10.2% in those with levels between 11.6 and 30 μmol/L, and 21.4% in those with levels < 11.6 μmol/L.

Assuming a dominant inheritance model, statistically significant associations were also found for the NPC1 rs1805081 and SCARB1 rs4238001 SNPs (*p* = 0.039 and *p* = 0.023, respectively). In the case of the recessive model, significant differences were observed for CD636 rs1761667 and rs1527479 SNPs (*p* = 0.001 and *p* = 0.004, respectively). It is interesting to note that for both SNPs, even when statistical significance was not reached, the frequency of the rare allele increased progressively from the group with the highest vitamin E levels to the group with intermediate levels, until it reached its highest values in the group with levels below 11.6 μmol/L (Table 3).

Notably, the allele frequencies of the CD636 rs1761667 SNP of the low-vitamin-E group (G = 43.1%, A = 56.9%) were more like the frequencies reported in the 1000 Genomes Project (https://www.internationalgenome.org/, 20 December 2024) for European populations (G = 47.3%, A = 52.7%—Supplementary Table S1) than those of the high-vitamin-E group (G = 54.1%, A = 45.9%). The opposite was found for the CD36 rs1527479 SNP, where the allele frequencies of the general European population (C = 53.6%, T = 46.4%) were closer to those of the group with high vitamin E levels (C = 52.4%, T = 47.6%) than those of the group with lower serum concentrations (C = 43.3%, T = 56.7%). The comparison of the allele distribution between the patient groups and the European population revealed interesting information for the APOE rs7412 SNP. In this case, the differences reached statistical significance and the frequencies in the general population (C = 93.7%, T = 6.3%) were intermediate compared to the high-vitamin-E group (C = 97.7%, T = 2.3%, *p* = 0.033) and the low-vitamin-E group (C = 88.2%, T = 11.8%, *p* = 0.035). The difference was even more pronounced when comparing the general population with the group with vitamin E levels below 11.6 μmol/L, where the allele T frequency was very high (C = 78.6%, T = 21.4%, *p* = 0.022). This trend was also confirmed at the genotype level (European population: CC = 87.7%, CT = 12.1%, TT = 0.2%; patients with vitamin E levels > 30 μmol/L: CC = 95.4%, CT = 4.6%, TT = 0.0%; patients with levels between 11.6 and 30 μmol/L: CC = 81.8%, CT = 15.9%, TT = 2.3%; patients with levels <11.6 μmol/L: CC = 71.4%, CT = 14.3%, TT = 14.3%; *p* < 0.0001).

A comparison of vitamin E levels before and after surgery was made to assess the response to vitamin E supplementation. The delta value was calculated for each patient as the difference between the vitamin E levels one year after surgery and before surgery, and the possible association with the SNPs studied was evaluated. The results are summarized in Table 4. A significant association was found between the APOE rs7412 SNP and a marked decrease in vitamin E levels after surgery, assuming additive (*p* = 0.019) and dominant (*p* = 0.001) models. In the latter case, the mean decrease went from −5.01 in the presence of the common allele C to −14.06 in the presence of the rare allele T. A similar trend was observed for the ABCA1 rs4149314 SNP. Again, significant differences were found for additive and dominant models (*p* = 0.032 and *p* = 0.005, respectively), with the rare allele being associated with a greater decrease in vitamin E levels.

For the other SNPs, significant associations were found for SCARB1 rs4238001 and rs11057830, assuming the recessive model (*p* = 0.021 and *p* = 0.016, respectively). For rs11057830, the rare allele was associated with a significant decrease in vitamin levels after surgery. Conversely, for the rs4238001, the rare allele was associated with an increase in mean vitamin E levels, with postoperative levels exceeding preoperative levels. A similar but less pronounced trend was also found for the APOA5 rs3135506 SNP, assuming a dominant model (*p* = 0.008), with a weaker decrease in the presence of the rare allele.

After analyzing each polymorphism separately, the statistical analysis was performed evaluating the haplotype combinations for the CD36 and APOE SNPs. While no significant associations were found for the CD36 haplotypes (Supplementary Table S2), the results for the APOE were of particular interest. APOE haplotypes of the rs429358 and rs7412 SNPs (defined as ε2, ε3 and ε4 haplotypes) produce different protein isoforms that interact differently with specific lipoprotein receptors and are considered risk factors for several diseases [7]. As shown in Table 5, in the group of patients with vitamin E deficiency (serum concentrations < 11.6 μmol/L), the frequency of the haplotype ε2, corresponding to the combination of the major variant rs429358 and the minor variant rs7412 (T-T), was increased compared to the other two groups. The difference was statistically significant when considering the distribution of the three possible haplotypes (*p* = 0.002), and the significance increased when the haplotype ε2 was compared with the other two groups (*p* = 0.001). A similar trend was observed for the correlation with the delta value of vitamin E concentrations. In this case, the ε2 haplotype was associated with a strong mean decrease in vitamin E levels (delta = −13.75), which was greater than for the ε3 and ε4 haplotypes (delta = −5.51); this was found to be statistically significant (*p* = 0.021) (Table 5).

## 3. Discussion

Today, bariatric surgery is considered the most effective treatment strategy that results in significant and sustained long-term weight loss and amelioration/remission of obesity-related comorbidities [2,8]. Malabsorptive and mixed procedures lead to greater weight loss compared to the other techniques, but they have been associated with an increased risk of early complications and malnutrition including vitamin E deficiency. Severe vitamin E deficiency is associated with neuronal disorders, impaired immune response, hemolytic anemia, and oxidative-based disorders [9]. Therefore, these patients are constantly supplemented; however, vitamin E did not always return to optimal serum concentrations in some cases. In this article, we highlighted the role of polymorphisms in genes involved with vitamin E transport and availability in reaching appropriate supplementation after bariatric surgery. We selected seven genes and 14 different SNPs, namely APOB rs1713222 and rs1042031; APOA5 rs662799 and rs3135506; APOE rs429358 and rs7412; NPC1 rs1805081; CD36 rs1761667 and rs1527479; SCARB1 rs4238001 and rs11057830; ABCA1 rs4149297, rs11789603 and rs4149314. Among all the SNPs, APOE rs7412 always appeared to be significantly correlated with vitamin E levels (at genotype and allele levels), considering the cut off at either 30 μmol/L or 11.6 μmol/L being the threshold required to have a beneficial effect on human health or the functional deficiency threshold, respectively. The APOE gene is located on chromosome 19 (19q13.32) and encodes for the major apoprotein of the chylomicron essential for the normal catabolism of triglyceride-rich lipoprotein constituents. The protein is also essential for vitamin E transport. Together with the APOE rs429358, the rs7412 polymorphism was mainly associated with plasma α-tocopherol, low-density lipoprotein cholesterol (LDL-C), and plasma total antioxidant capacity [10,11]. In our cohort of patients undergoing bariatric surgery, the rare T allele in the rs7412 increases while the vitamin E level decreases, indicating that in heterozygous or rare homozygous patients, vitamin E transport is impaired, and that, even if correctly supplemented, in these patients, the target concentration cannot be reached (or is achieved with more difficulty). This was evident also considering the delta between post- and pre-surgery vitamin E concentrations.

The two APOE SNPs, rs429358 and rs7412, were used to identify three main APOE haplotypes: ε2, ε3, and ε4. The ε3 isoform represents the most common haplotype in the population, ranging from 40 to 90%, and it is considered neutral [5], whereas ε2 is linked to an inactivation of the receptor-binding region and ε4 affects the lipid-binding site of the protein finally decreasing vitamin E transport. In particular, APOE-ε4 subjects have been shown to have an increased demand of vitamin E [5]. In bariatric patients, in the group with vitamin E deficiency (serum concentrations < 11.6 μmol/L), the frequency of the haplotype ε2 (T-T) was significantly increased compared to the other two groups, indicating that vitamin E deficiency in this group of patients can be linked to an impaired bound between vitamin E and its receptor. Conducting in vitro or in vivo studies could help confirm this hypothesis and clarify the mechanism that underlies this observation. For the other analyzed genes, assuming a dominant inheritance model, statistically significant associations were also found for the SCARB1 rs4238001 whereas in the case of the recessive model, significant differences were observed for CD36 rs1761667 and rs1527479 SNPs. Both these genes are involved with vitamin E bioavailability. In detail, the CD36 gene is located on the q11.2 region of chromosome 7 and encodes for a membrane glycoprotein expressed on the apical sides of enterocytes where interacts with fatty acids facilitating chylomicron assembly and vitamin E uptake [12]. For SNP rs1761667, the rare A allele has been associated with a reduced expression of the CD36 transcript [13] having an impact on metabolic syndrome development, HDL metabolism and vitamin E absorption. SCARB1 gene is located on chromosome 12q24.31 and encodes for a multi-ligand membrane receptor which mediates cholesterol transfer to and from HDL and vitamin E uptake at hepatic level [14]. For SNP rs4238001, the rare allele (T) has been associated with SCARB1 protein degradation and lower protein levels [5] possibly involved with the vitamin E uptake impairment observed in our patients. If we consider the difference in vitamin E level post and pre bariatric surgery, for the rs4238001 the rare allele was associated with an increase in mean vitamin E levels, with postoperative levels exceeding preoperative ones possibly indicating a positive effect on vitamin E availability. On the contrary, the rare allele of SCARB1 rs11057830, was associated with a significant decrease in vitamin levels after surgery as reported also in the literature [15]. Finally, considering the delta between post and pre surgery, the rare allele in the rs4149314 of the ABCA1 gene was also linked to decreased vitamin E levels both in the additive or dominant model. The ABCA1 protein is a transporter expressed in several tissues like the liver, intestine, and macrophages with a role in cholesterol transport, lipid metabolism, and vitamin E bioavailability and distribution [16].

This study has both limitations and strengths that are worth noting. A key strength lies in the thorough clinical characterization and follow-up of all participants. However, factors such as dietary habits and lifestyle were not included in this analysis. It is important to note that all patients were regularly supplemented with Baritrifast, a dietary supplement specifically designed to support nutritional needs in this population. Baritrifast contains a blend of essential vitamins, minerals, specific for the needs of bariatric patients. Adherence to the supplementation regimen and prescribed therapy was closely monitored during each medical visit, where patients were systematically assessed for compliance through detailed interviews. Moreover, the sample size may not be sufficient to achieve a high statistical power, potentially limiting the ability to generalize the results to the broader population. In addition, due to the low number of patients the interaction between genetic and biochemical data has been only partially addressed, with limited consideration of multifactorial effects. Future replication studies with a larger group of obese patients would be beneficial to more accurately assess potential associations between vitamin E concentration, SNPs, and these lifestyle variables.

## 4. Materials and Methods

### 4.1. Subjects

This study included an Italian cohort composed of 140 patients with a body mass index (BMI) > 30 kg/m^2^ (BMI at diagnosis: 48.65 ± 5.03) who underwent bariatric surgery (malabsorptive surgeries were not included). The patients, 26 men (18.6%) and 114 women (81.4%), had a mean age at diagnosis of 39 ± 13 years. Inclusion criteria were BMI ≥ 40 kg/m^2^ or ≥ 35 kg/m^2^ associated with treated or untreated dyslipidemia (cholesterol LDL > 140 mg/dL and/or total cholesterol > 200 mg/dL), hypertension or type 2 Diabetes in patients who had previously failed multiple lifestyle interventions like diet and physical activity. The following were assessed at screening: glycaemic control parameters (fasting glucose, insulin and glycated hemoglobin), liver function and cardiometabolic biomarkers (lipid assessment, uric acid, pulse rate and blood pressure). To exclude endocrine causes of obesity, measurement of thyroid-stimulating hormone (TSH), free triiodothyronine (FT3), free thyroxine (FT4), cortisol and adrenocorticotropic hormone (ACTH) levels at baseline and after administration of 1 mg dexamethasone overnight was performed. Biochemical parameters are summarized in Table 6.

Each patient gave written informed consent, and this study was performed in accordance with the ethical standards laid down in the 1964 Declaration of Helsinki and its later amendments, and was approved by our local Ethical Committee (protocol n. 21539).

### 4.2. Vitamin E Assessment

Serum vitamin E concentrations were assessed using the spectrophotometric method previously described by Rutkowski and Grzegorczyk, 2007 [17].

### 4.3. DNA Extraction and PCR

Genomic DNA (gDNA) was extracted from peripheral blood leukocytes using the salting-out method and stored at −20 °C until PCR analysis. DNA purity and its concentration were assessed by Nanodrop One (Thermo Scientific, Milan, Italy). Selected SNPs (Supplementary Table S3) were amplified by end-point PCR using 200 nM specific primers for 35 cycles. Annealing temperatures and PCR conditions are reported in Supplementary Table S3.

### 4.4. Restriction FragmentLength

Analysis of six SNPs was performed by restriction fragment length polymorphism analysis using the following restriction endonucleases: HhaI for CD36 rs17161667; AlwnI for CD36 rs1527479; AluI for SCARB1 rs 4238001; NcoI for NPC1 rs 1805081; Bsu36I for APOB rs 1713222; EcoRI for APOB rs 1042031. Restriction was carried out at 37 °C O/N and then visualized on an agarose gel (3%).

### 4.5. Denaturing HPLC(DHPLC) and Sequencing

For some SNPs (ABCA1 rs11789603; ABCA1 rs4149314; APOA5 rs662799 and APOA5 rs3135506), PCR products were analyzed by denaturing high-performance liquid chromatography (DHPLC) (Transgenomic, Inc., Omaha, NE, USA) at specific temperatures (Supplementary Table S4) alone and mixed with an approximately equimolar volume of a wild-type sample to define the genotype of homoduplex samples. To confirm the presence/absence of variants, a subset of samples was directly sequenced by Sanger sequencing using the Thermo Fisher 3500 Series Genetic Analyzer (Thermo Scientific, Milan, Italy).

### 4.6. Statistical Analysis

All statistical analyses were carried out by using the software package SPSS v13.0. *p* < 0.05 was considered statistically significant. Haplotype frequencies and association statistics for polymorphisms were constructed using PHASE version 2 software [18].

Interaction with polymorphisms was tested by χ2 analysis at genotype, allele, and haplotype levels.

To evaluate the association between gene polymorphisms and serum vitamin E concentrations, patients were first stratified into two different groups (<30 μmol/L, >30 μmol/L) and then into three different groups (<11.6 μmol/L, 11.6–30 μmol/L, >30 μmol/L). Association analyses were performed using χ2 or Fischer’s exact test. To assess the response to vitamin E supplementation, the delta value between vitamin E levels one year after surgery and before surgery was calculated for each patient. The association with delta was performed using the -Mann–Whitney U test.

For the analysis of the association of APOE haplotypes with vitamin E levels and delta values, the haplotypes were APOE ε2, corresponding to the major variant rs429358 and the minor variant rs7412 (T-T), APOE ε3, corresponding to the more frequent variants for both alleles (T-C), and APOE ε4, composed of the minor variant rs429358 and the major variant rs7412 (CC). Associations with haplotypes were also assessed for CD36 SNPs (rs1761667 and rs1527479; possible haplotypes: A-C, G-T, G-C, A-T). Association analyses were performed using the χ2 test for association with vitamin E levels and the -Kolmogorov–Smirnov test or -Mann–Whitney U test for association with delta values.

## 5. Conclusions

In conclusion, our study describes several SNPs that are associated with impaired vitamin E concentration after bariatric surgery; this study suggests that the association of multiple variants should be taken into consideration to better assess the risk of malabsorptive phenotype development.

## Figures and Tables

**Table 1:** Differences at genotype and allele levels after having stratified patients according to the cut off of 30 μmol/L.

SNP	>30 (%)			<30 (%)			*p* Value	Odds Ratio (95% Confidence Interval)
**APOB rs1713222 G/A**	**GG**	**AG**	**AA**	GG	AG	AA		
Genotype	73.6	24.1	2.3	66.0	34.0	0.0	0.321	
Allele	G 85.6		A 14.4	G 83.0		A 17.0	0.609	1.219 (0.630–2.360)
**APOB rs1042031 C/T**	CC	CT	TT	CC	CT	TT		
Genotype	63.1	31.0	6.0	62.3	35.8	1.9	0.524	
Allele	C 78.6		T 21.4	C 80.2		T 19.8	0.879	0.906 (0.496–1.656)
**APOA5 rs3135506 G/C**	GG	GC	CC	GG	GC	CC		
Genotype	81.6	17.2	1.1	88.7	11.3	0.0	0.668	
Allele	G 90.2		C 9.8	G 94.3		C 5.7	0.267	0.554 (0.211–1.453)
**NPC1 rs1805081 T/C**	TT	TC	CC	TT	TC	CC		
Genotype	48.8	39.5	11.6	46.2	44.2	9.6	0.848	
Allele	T 68.6		C 31.4	T 68.3		C 31.7	1.000	1.016 (0.602–1.715)
**CD36 rs1761667 G/A**	GG	GA	AA	GG	GA	AA		
Genotype	25.9	56.5	17.6	19.6	47.1	33.3	0.135	
Allele	G 54.1		A 45.9	G 43.1		A 56.9	0.103	1.555 (0.948–2.549)
**CD36 rs1527479 C/T**	CC	CT	TT	CC	CT	TT		
Genotype	25.0	54.8	20.2	21.2	44.2	34.6	0.186	
Allele	C 52.4		T 47.6	C 43.3		T 56.7	0.170	1.442 (0.882–2.359)
**SCARB1 rs4238001 G/A**	GG	GA	AA	GG	GA	AA		
Genotype	73.3	26.7	0.0	72.0	26.0	2.0	0.568	
Allele	G 86.6		A 13.4	G 85.0		A 15.0	0.720	1.143 (0.566–2.309)
**SCARB1 rs11057830 G/A**	GG	GA	AA	GG	GA	AA		
Genotype	62.8	30.2	7.0	50.0	38.0	12.0	0.309	
Allele	G 77.9		A 22.1	G 69.0		A 31.0	0.113	1.143 (0.566–2.309)
**APOE rs429358 T/C**	TT	TC	CC	TT	TC	CC		
Genotype	79.3	20.7	0.0	76.5	23.5	0.0	0.831	
Allele	T 89.7		C 10.3	T 88.2		C 11.8	0.695	1.156 (0.532–2.509)
**APOE rs7412 C/T**	CC	CT	TT	CC	CT	TT		
Genotype	95.4	4.6	0.0	80.4	15.7	3.9	**0.012**	
Allele	C 97.7		T 2.3	C 88.2		T 11.8	**0.002**	**5.667 (1.776–18.078)**
**ABCA1 rs11789603 G/A**	GG	GA	AA	GG	GA	AA		
Genotype	94.3	5.7	0.0	96.2	3.8	0.0	0.710	
Allele	G 97.1		A 2.9	G 98.1		A 1.9	0.713	0.650 (0.124–3.411)
**ABCA1 rs4149297 A/G**	AA		AG	GG		AA	AG	GG
Genotype	77.9		22.1	0.0		84.3	15.7	0.0
Allele	A 89.0		G 11.0	A 92.2		G 7.8	0.530	0.685 (0.289–1.628)
**ABCA1 rs4149314 A/G**	AA	AG	GG	AA	AG	GG		
Genotype	79.8	17.9	2.4	86.3	13.7	0.0	0.546	
Allele	A 88.7		G 11.3	A 93.1		G 6.9	0.290	0.578 (0.234–1.427)

**Table 2:** Differences at genotype and allele levels after having stratified patients according to the cut off of 11.6 μmol/L.

SNP	>30 (%)			11.6–30 (%)			<11.6 (%)			*p* Value
**APOB rs1713222 G/A**	**GG**	**AG**	**AA**	**GG**	AG	AA	GG	AG	AA	
Genotype	73.6	24.1	2.3	66.7	33.3	0.0	62.7	37.5	0.0	0.555
Allele	G 85.6		A 14.4	G 83.3		A 16.7	G 81.3		A 18.8	0.756
**APOB rs1042031 C/T**	CC	CT	TT	CC	CT	TT	CC	CT	TT	
Genotype	63.1	31.0	6.0	60.0	37.8	2.2	75.0	25.0	0.0	0.827
Allele	C 78.6		T 21.4	C 78.9		T 24.1	C 87.5		T 12.5	0.958
**APOA5 rs3135506 G/C**	GG	GC	CC	GG	GC	CC	GG	GC	CC	
Genotype	81.6	17.2	1.1	86.7	13.3	0.0	100.0	0.0	0.0	0.710
Allele	G 90.2		C 9.8	G 93.3		C 6.7	G 100.0		C 0.0	0.431
**NPC1 rs1805081 T/C**	TT	TC	CC	TT	TC	CC	TT	TC	CC	
Genotype	48.8	39.5	11.6	48.9	42.2	8.9	14.3	71.4	14.3	0.412
Allele	T 68.6		C 31.4	T 70.0		C 30.0	T 50.0		C 50.0	0.322
**CD36 rs1761667 G/A**	GG	GA	AA	GG	GA	AA	GG	GA	AA	
Genotype	25.9	56.5	17.6	20.5	50.0	29.5	14.3	28.6	57.1	0.153
Allele	G 54.1		A 45.9	G 45.5		A 54.5	G 28.6		A 71.4	0.113
**CD36 rs1527479 C/T**	CC	CT	TT	CC	CT	TT	CC	CT	TT	
Genotype	25.0	54.8	20.2	22.2	46.7	31.1	14.3	28.6	57.1	0.252
Allele	C 52.4		T 47.6	C 45.6		T 54.4	C 28.6		T 71.4	0.170
**SCARB1 rs4238001 G/A**	GG	GA	AA	GG	GA	AA	GG	GA	AA	
Genotype	73.3	26.7	0.0	67.4	30.2	2.3	100.0	0.0	0.0	0.199
Allele	G 86.6		A 13.4	G 82.6		A 17.4	G 100.0		A 0.0	0.228
**SCARB1 rs11057830 G/A**	GG	GA	AA	GG	GA	AA	GG	GA	AA	
Genotype	62.8	30.2	7.0	46.5	39.5	14.0	71.4	28.6	0.0	0.379
Allele	G 77.9		A 22.1	G 66.3		A 33.7	G 85.7		A 14.3	0.088
**ABCA1 rs4149297 A/G**	AA	AG	GG	AA	AG	GG	AA	AG	GG	
Genotype	77.9	22.1	0.0	83.7	16.3	0.0	87.5	12.5	0.0	0.717
Allele	A 89.0		G 11.0	A 91.9		G 8.1	A 93.8		G 6.3	0.742
**APOE rs429358 T/C**	TT	TC	CC	TT	TC	CC	TT	TC	CC	
Genotype	79.3	20.7	0.0	79.5	20.5	0.0	57.1	42.9	0.0	0.379
Allele	T 89.7		C 10.3	T 89.8		C 10.2	T 78.6		C 21.4	0.395
**APOE rs7412 C/T**	CC	CT	TT	CC	CT	TT	CC	CT	TT	
Genotype	95.4	4.6	0.0	81.8	15.9	2.3	71.4	14.3	14.3	**0.008**
Allele	C 97.7		T 2.3	C 89.8		T 10.2	C 78.6		T 21.4	**0.002**
**ABCA1 rs11789603 G/A**	GG	GA	AA	GG	GA	AA	GG	GA	AA	
Genotype	94.3	5.7	0.0	97.8	2.2	0.0	87.5	12.5	0.0	0.308
Allele	G 97.1		A 2.9	G 98.9		A 1.1	G 93.8		A 6.3	0.311
**ABCA1 rs4149314 A/G**	AA	AG	GG	AA	AG	GG	AA	AG	GG	
Genotype	79.8	17.9	2.4	86.4	13.6	0.0	85.7	14.3	0.0	0.851
Allele	A 88.7		G 11.3	A 93.2		G 6.8	A 92.9		G 7.1	0.492

**Table 3:** Dominant and recessive model.

SNP *Dominant Model*	>30 (%)	11.6–30 (%)	<11.6 (%)	*p* Value
**APOB rs1713222 G/A**				
GG	73.6	66.7	62.3	0.363
AA + GA	26.4	33.3	37.5	
**APOB rs1042031 C/T**				
CC	63.1	60.0	75.0	0.569
TT + CT	36.9	40.0	25.0	
**APOA5 rs3135506 G/C**				
GG	81.6	86.7	100.0	0.110
CC + GC	18.4	13.3	0.0	
**NPC1 rs1805081 T/C**				
TT	48.8	48.9	14.3	**0.039**
CC + TC	51.2	51.1	85.7	
**CD36 rs1761667 G/A**				
GG	25.9	20.5	14.3	0.517
AA + GA	74.1	79.5	85.7	
**CD36 rs1527479 C/T**				
CC	25.0	22.2	14.3	0.689
TT + CT	75.0	77.8	85.7	
**SCARB1 rs4238001 G/A**				
GG	73.3	67.4	100.0	**0.023**
AA + GA	26.7	32.6	0.0	
**SCARB1 rs11057830 G/A**				
GG	62.8	46.5	71.4	0.028
AA + GA	37.2	53.5	28.6	
**ABCA1 rs4149297 A/G**				
AA	77.9	83.7	87.5	0.523
GG + AG	22.1	16.3	12.5	
**APOE rs429358 T/C**				
TT	79.3	79.5	57.1	0.172
CC + TC	20.7	20.5	42.9	
**APOE rs7412 C/T**				
CC	95.4	81.8	71.4	**<0.0001**
TT + CT	4.6	18.2	28.6	
**ABCA1 rs11789603 G/A**				
GG	94.3	97.8	87.5	0.115
AA + GA	5.7	2.2	12.5	
**ABCA1 rs4149314 A/G**				
AA	79.8	86.4	85.7	0.424
GG + AG	20.2	13.6	14.3	
**SNP** *recessive model*	**>30 (%)**	**<30 (%)**	***p* value**	**OR**
**APOB rs1713222 G/A**				
AA	2.3	0.0	0.301	0.977 (0.955–1.000)
GG + GA	97.7	100.0		
**APOB rs1042031 C/T**				
TT	6.0	1.9	0.137	0.304 (0.065–1.415)
CC + CT	94.0	98.1		
**APOA5 rs3135506 G/C**				
CC	1.1	0.0	0.528	0.989 (0.973–1.004)
GG + GC	98.9	100.0		
**NPC1 rs1805081 T/C**				
CC	11.6	9.6	0.692	0.809 (0.363–1.802)
TT + TC	88.4	90.4		
**CD36 rs1761667 G/A**				
AA	17.6	33.3	**0.003**	**2.333 (1.319–4.126)**
GG + GA	82.4	66.7		
**CD36 rs1527479 C/T**				
TT	20.2	34.6	**0.008**	**2.087 (1.201–3.624)**
CC + CT	79.8	65.4		
**SCARB1 rs4238001 G/A**				
AA	0.0	2.0	0.134	1.020 (0.992–1.049)
GG + GA	100.0	98.0		
**SCARB1 rs11057830 G/A**				
AA	7.0	12.0	0.185	1.818 (0.784–4.217)
GG + GA	93.0	88.0		
**ABCA1 rs4149297 A/G**				
GG	0.0	0.0	/	/
AA + AG	100.0	100.0		
**APOE rs429358 T/C**				
CC	0.0	0.0	/	/
TT + TC	100.0	100.0		
**APOE rs7412 C/T**				
TT	0.0	3.9	**0.018**	**1.041 (1.001–1.082)**
CC + CT	100.0	96.1		
**ABCA1 rs11789603 G/A**				
AA	0.0	0.0	/	/
GG + GA	100.0	100.0		
**ABCA1 rs4149314 A/G**				
GG	2.4	0.0	0.301	0.976 (0.953–1.000)
AA + AG	97.6	100.0		

**Table 4:** Comparison of vitamin E levels before and after surgery.

SNP	Model	Δ (Means)		*p* Value
**APOB rs1713222 G/A**	ADD	G −5.65	A −7.22	0.964
	DOM	G −5.15	A −7.68	0.242
	REC	G −6.01	A 0.95	0.055
**APOB rs1042031 C/T**	ADD	C −6.20	T −5.53	0.857
	DOM	C −6.21	T −5.81	0.849
	REC	C −6.19	T −2.94	0.229
**APOA5 rs3135506 G/C**	ADD	G −6.02	C −4.16	0.071
	DOM	**G −6.18**	**C −4.16**	**0.008**
	REC	G −5.89	C none	/
**NPC1 rs1805081 T/C**	ADD	T −6.47	C −5.22	0.713
	DOM	T −7.01	C −5.21	0.460
	REC	T −6.18	C −5.28	0.939
**CD36 rs1761667 G/A**	ADD	G −5.11	A −6.59	0.250
	DOM	G −4.13	A −6.38	0.227
	REC	G −5.41	A −7.34	0.124
**CD36 rs1527479 C/T**	ADD	C −6.23	T −5.28	0.519
	DOM	C −5.94	T −5.69	0.684
	REC	C −6.33	T −4.00	0.273
**SCARB1 rs4238001 G/A**	ADD	G −5.70	A −7.92	0.507
	DOM	G −5.08	A −8.52	0.129
	REC	**G −6.16**	**A 11.30**	**0.021**
**SCARB1 rs11057830 G/A**	ADD	G −5.29	A −7.56	0.066
	DOM	G −5.13	A −6.87	0.059
	REC	**G −5.39**	**A −11.01**	**0.016**
**ABCA1 rs4149297 A/G**	ADD	A −5.84	G −6.05	0.897
	DOM	A −5.81	G −6.05	0.844
	REC	A −5.87	G none	/
**APOE rs429358 T/C**	ADD	T −5.93	C −6.18	0.990
	DOM	T −5.89	C −6.18	0.983
	REC	T −5.69	C none	/
**APOE rs7412 C/T**	ADD	**C −5.51**	**T −13.75**	**0.019**
	DOM	**C −5.01**	**T −14.06**	**0.001**
	REC	C −5.92	T −10.10	0.322
**ABCA1 rs11789603 G/A**	ADD	G −5.98	A −1.86	0.276
	DOM	G −6.06	A −1.86	0.112
	REC	G −5.89	A none	/
**ABCA1 rs4149314 A/G**	ADD	**A −5.52**	**G −9.74**	**0.032**
	DOM	**A −5.233**	**G −9.02**	**0.005**
	REC	A −5.76	G −18.00	0.087

ADD: additive model; DOM: dominant model; REC: recessive model.

**Table 5:** Frequencies of APOE haplotypes according to vitamin E levels and mean variation one year after surgery.

APOE Haplotype	>30 (%)	11.6–30 (%)	<11.6 (%)	*p* Value	Δ (Means)	*p* Value
**ε2**	2.3	10.5	21.4	**0.002**	−13.75	0.069
**ε3**	88.4	80.2	57.1		−5.479	
**ε4**	9.3	9.3	21.4		−6.14	
**ε2**	2.3	10.5	21.4	**0.001**	−13.75	**0.021**
**ε3 + ε4**	97.7	89.5	78.6		−5.56	

**Table 6:** Biochemical parameters of the subjects.

Parameter	
Number of subjects (n)	140
Gender (M:F)	26:114
Age at diagnosis (years)	39 ± 13
BMI at diagnosis	48.65 ± 5.03
Waist circumference (cm)	125.9 ± 15.5
HDL cholesterol (mg/dL)	42.7 ± 10.6
Triglycerides (mg/dL)	189.9 ± 113
Glycemia (mg/dL)	125.2 ± 54.5
Insulin (mUI/L)	24.7 ± 29.4
HOMA-IR	6.9 ± 8.5
Hypertension (%)	45
Steatosis (%)	81
Dyslipidemia (%)	47.2

## Data Availability

Data are contained within the article and Supplementary Materials.

## Supplementary Materials

The following supporting information can be downloaded at: https://www.mdpi.com/article/10.3390/ijms26020651/s1.
