# Supplementary material for: Impact of Genetic Variants on Vitamin E Levels in an Italian Cohort of Bariatric Surgery Patients: A Focus on SNPs Involved with Transport and Bioavailability
Source: Int J Mol Sci. 2025 Jan 14;26(2):651. doi: 10.3390/ijms26020651 (PMC11765794; doi:10.3390/ijms26020651)
Supplement: Supplementary file 1 [file ijms-26-00651-s001.zip › ijms-3403054-supplementary.pdf]

International Journal of Molecular Sciences

MDPI

Supplementary Table S1: Frequencies in the European Population for each SNPs

SNP	Frequencies in European Population % (number of subjects)		
**APOB rs1713222 G/A**	GG	AG	AA
Genotype	65.0 (327)	32.4 (163)	2.6 (13)
Allele	G 81.2 (817)		A 18.8 (189)
**APOB rs1042031 C/T**	CC	CT	TT
Genotype	62.4 (314)	32.6 (164)	5.0 (25)
Allele	C 78.7 (792)		T 21.3 (214)
**APOA5 rs3135506 G/C**	GG	GC	CC
Genotype	87.1 (438)	12.3 (62)	0.6 (3)
Allele	G 93.2 (938)		C 6.8 (68)
**NPC1 rs1805081 T/C**	TT	TC	CC
Genotype	37.6 (189)	48.1 (242)	14.3 (72)
Allele	T 61.6 (620)		C 38.4 (386)
**CD36 rs1761667 G/A**	GG	GA	AA
Genotype	23.5 (118)	47.7 (240)	28.8 (145)
Allele	G 47.3 (476)		A 52.7 (530)
**CD36 rs1527479 C/T**	CC	CT	TT
Genotype	29.6 (149)	47.9 (241)	22.5 (113)
Allele	C 53.6 (539)		T 46.4 (467)
**SCARB1 rs4238001 G/A**	GG	GA	AA
Genotype	78.5 (395)	19.7 (99)	1.8 (9)
Allele	G 88.4 (889)		A 11.6 (117)
**SCARB1 rs11057830 G/A**	GG	GA	AA
Genotype	69.4 (349)	28.2 (142)	2.4 (12)

Allele	G 83.5 (840)		A 16.5 (166)
**ABCA1 rs4149297 A/G**	AA	AG	GG
Genotype	80.5 (405)	18.9 (95)	0.6 (3)
Allele	A 90.0 (905)		G 10.0 (101)
**APOE rs429358 T/C**	TT	TC	CC
Genotype	71.2 (358)	26.6 (134)	2.2 (11)
Allele	T 84.5 (850)		C 15.5 (156)
**APOE rs7412 C/T**	CC	CT	TT
Genotype	87.7 (441)	12.1 (61)	0.2 (1)
Allele	C 93.7 (943)		T 6.3 (63)
**ABCA1 rs11789603 G/A**	GG	GA	AA
Genotype	82.1 (413)	17.1 (86)	0.8 (4)
Allele	G 90.7 (912)	A 9.3 (94)	
**ABCA1 rs4149314 A/G**	AA	AG	GG
Genotype	81.3 (409)	18.3 (92)	0.4 (2)
Allele	A 90.5 (910)		G 9.5 (96)

* 1000 Genomes Project (11th October 2024)

Supplementary Table S2: CD36 haplotypes

CD36 Haplotype	>30 (%)	11.6-30 (%)	<11.6 (%)	*p* value
**A-C**	45.1	52.3	71.4	0.156
**G-T**	53.0	43.0	28.6	
**G-C**	1.8	2.3	0.0	
**A-T**	0.0	2.3	0.0	

CD36 Haplotype	Δ (means)	*p* value
**A-C**	-6.41	0.211
**G-T**	-5.06	
**G-C**	-3.64	
**A-T**	-15.40	

Supplementary Table S3: SNPs, primers and PCR conditions for each SNPs

SNP	Primer Sequence	Product Size (bp)	Annealing Temperature (°C)	MgCl Concentration
APOB rs1713222	For:TATCCATCCAGCTATCCGGC<br>Rev:CGACCTCTCCAAAGACCTGT	358 bp	60 °C	2.5 mM
APOB rs1042031	For: TGGGTTTATCAAGGGGCCAT<br>Rev: ACCGTCCCTACCTCCCTTAT	341 bp	60 °C	2.5 mM
APOA5 rs662799	For: GGAGTGGGTGTGTCATCAGA<br>Rev: CGGCAAGATGGACAGTGTTC	337 bp	60 °C	2.5 mM
APOA5 rs3135506	For: TCTCCGACCCTGACTTCAAC<br>Rev: CTCCTTTCCTCTGTCCCAGC		60 °C	2.5 mM
NPC1 rs1805081	For: AGGACGAAGCAGCAAAACAT<br>Rev: GGGGCCACAGACAATAGAG	283 bp	60 °C	2.5 mM
CD36 rs1761667	For: TGCAACTTCCCTGAAAACCA<br>Rev: AGAGTTTTCATGAAGCTTCCCG	400 bp	60 °C	2.5 mM
CD36 rs1527479	For: TCCCTGGTTTCTTCAGTGGT<br>Rev: CCCCAGTGTCACATTCCAGT	388 bp	60 °C	2.5 mM
SCARB1 rs4238001	For: GCATAAAACCACTGGCCACC<br>Rev: AAGGACCTGCTGCTTGATGA	299 bp	60 °C	1.5 mM
ABCA1 rs4149297	For: GACCTAGAGAGTGCTGGACC<br>Rev: TAGAACAGTCCCAACAGCGT	387 bp	60 °C	2.5 mM
APOE rs429358 / rs7412	For: GGCACGGCTGTCCAAGGA<br>Rev: GGCAGTGTACCAGGCCGGGGC		60 °C	2.5 mM
ABCA1 rs11789603	For: AGCCCACTTCATCTTACCGT<br>Rev: CTTCATGCCAAGTCTGTCCG	309 bp	60 °C	2.5 mM
ABCA1 rs4149314	For: GCCTCAGATGACCTTTCCCA<br>Rev: ACAGGAAAAGAATGTCACCCC	336 bp	60 °C	2.5 mM

Supplementary Table S4. DHPLC conditions for each SNPs

**DHPLC conditions**	
SNP	Temperature (°C)
APOA5 rs3135506	62.8 °
ABCA1 rs4149314	57.7 °
ABCA1 rs11789603	57.3°
APOA5 rs662799	60.8 °C
